# The Case for a Medication Safety Curriculum for Residents in Pediatric Settings

**DOI:** 10.1097/pq9.0000000000000859

**Published:** 2025-12-08

**Authors:** Haley Davis, Julianna Lau, Lisa M. Rickey

**Affiliations:** From the * Department of Pediatrics, Columbia University Irving Medical Center, New York-Presbyterian Morgan Stanley Children’s Hospital, New York, N.Y.; † Department of Pediatrics, Boston Children’s Hospital, Boston, Mass.; ‡ Department of Pediatrics, Harvard Medical School, Boston, Mass.

Consider this real-life scenario: an adult emergency medicine trainee treats a toddler with gastroenteritis and orders maintenance intravenous fluids without dextrose. Although safely tolerated in adults, children require dextrose in intravenous fluids to prevent hypoglycemia. Subsequently, the child becomes lethargic and has a seizure. Her test results indicate she is severely hypoglycemic and requires inpatient admission for further treatment and stabilization.

Reducing medication-related harm is a key priority in patient safety.^1^ Medication errors in children are common, costly, and pose a significant risk for patient harm.^2–4^ Health systems have leveraged safety redundancies (eg, computerized provider order entry systems, automated dose calculation, and dose checking), high-reliability principles, and human factors design to identify and intercept medication errors before they reach patients. Numerous system-based interventions have been effective yet insufficient in achieving a zero-harm goal.^5,6^ We propose that structured education for early career clinicians may be critical to drive further improvements in medication safety outcomes, particularly when integrated as 1 component of broader institutional harm-reduction strategies.

There is no published data or available guidance on which fundamental topics should be included in pediatric medication safety education, which introduces variability in the core educational content that residents receive. In the current state of medication safety education, many medical trainees learn essential principles in medication safety through informal teaching and feedback from vigilant pharmacists or supervising clinicians. Although situational feedback is a valuable educational tool, it can be inconsistent, insufficient, and is a *reactive* rather than a *proactive* approach to patient safety. Therefore, there is a clear need for a standardized medication safety curriculum in graduate medical education. This is of paramount importance for pediatric residents, with generalized benefit for residents of all specialties who will provide care for children during their training.

A systematic review of interventions to reduce pediatric medication errors identified multiple areas for improvement, including the need for enhanced training for medical professionals.^7^ Pediatric trainees represent a high-yield population for focused interventions, with nearly 4,000 pediatric trainees working in US hospitals annually. Residents are typically the frontline providers who complete medication reconciliations, order medications, and navigate the complexities of prescribing. Hectic schedules and frequently changing clinical settings further compound the risk of error. On average, a pediatric resident enters more than 4,300 electronic orders annually.^8^ One study found that 8.4% of medication orders placed by first-year doctors contained an error.^9^ Interventions that are implemented early in training related to knowledge, skills, and attitudes in medication safety have the potential to be carried forward and sustained throughout a career. Data from the critical care literature demonstrate that structured medication safety education leads to a reduction in medication errors.^10^ However, there is currently no explicit requirement from the Accreditation Council for Graduate Medical Education for training programs to include a pediatric medication safety curriculum. Although there are requirements for trainees to understand medication side effects and identify adverse events, there is little guidance on how trainees should acquire these skills.

**Fig. 1.**
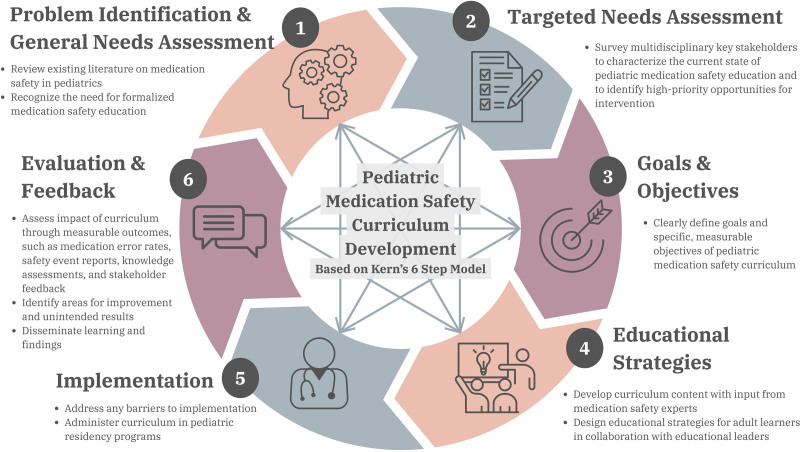
Proposed process for pediatric medication safety curriculum design following Kern’s 6 steps.

Medication safety is a collaborative effort involving multiple stakeholders, and an interdisciplinary approach to developing a medication safety curriculum is essential. Quality improvement methodologies should be used throughout the development of a medication safety curriculum, from analyzing the current state of resident education to understand the problem, identifying opportunities for change, designing and implementing targeted educational interventions, and continually measuring impact with defined outcomes. This process mirrors Kern’s 6 steps for curriculum development in medical education (Fig. 1).^11^ According to Kern’s model, clarification of the problem begins with a needs assessment of key stakeholders, ideally including residents, pharmacists, nurses, patient safety leaders, educational leaders, and caregivers. The needs assessment should explore the most important topics in medication safety, existing and perceived knowledge and training gaps, and the preferred method of delivery from residents. Findings from this needs assessment will inform the key topics to be included in the curriculum. Subsequent steps in curriculum development include setting goals, defining educational strategies, implementing them, and evaluating the results. Throughout the change process, it is important to systematically assess the impact of the curriculum, including any unintended consequences. Outcomes should measure both learner-related impact (eg, resident feedback, knowledge assessments) and impact on patients and systems (eg, error rates, safety event reports). The results can be used to inform future cycles of curricular improvement.

Although pediatric residencies are the natural starting point for this initiative, the underlying medication safety principles are broadly applicable across many specialties, as residents from nearly all medical specialties rotate in pediatric settings during their training. Pediatric programs can lead initial iterations of curriculum design and share results using the Standards for QUality Improvement Reporting Excellence education guidelines, which provide a framework for reporting quality improvement work in medical education.^12^ By using a standardized framework to report findings, lessons learned can be disseminated beyond local contexts and adapted for other training programs that provide pediatric care, such as family medicine, emergency medicine, and surgical specialties.

Notably, educational interventions alone are insufficient to achieve highly reliable outcomes. Education is a complementary and essential component of a system-wide safety strategy. Robust medication safety programs require a multifaceted approach, which includes education, in addition to strategies such as technological advancements to reduce errors, systems for managing high-risk medications, and teamwork training.^5,7^ Even within residency programs, this curriculum represents 1 facet of a broader approach to medication safety, which may include integration of pharmacists on rounds, participation in medication error event reviews, and inclusion of residents as key stakeholders in system-wide medication safety efforts.^5,7^ Incorporating the curriculum within an organization’s larger initiatives can promote a more comprehensive and sustainable safety culture.

Ultimately, given the frequency of pediatric medication errors and their potential for harm, it is incongruent that no formal educational standard exists for residencies. Therefore, we advocate for the development of a comprehensive pediatric medication safety curriculum to capitalize on an existing opportunity to mitigate medication-related harm. By using a multidisciplinary approach rooted in quality improvement and curriculum development methodologies, we can integrate medication safety into residency training and equip future generations of providers to deliver the highest quality care to pediatric patients.
